# Using feeding regime as a microbial selective pressure to optimise biogas production and digestate sanitisation from slurry-based anaerobic digestion

**DOI:** 10.1186/s40793-026-00902-x

**Published:** 2026-05-22

**Authors:** S. Nolan, A. Trego, N. Waters, C. Thorn, O. Fenton, K. G. Richards, V. O’Flaherty, U. Z. Ijaz, F. Abram

**Affiliations:** 1 School of Biological and Chemical Sciences, Ryan Institute, University of Galway, Galway, Ireland; 2 Teagasc, Environmental Research Centre, Johnstown Castle, Wexford, Ireland; 3 Information and Computational Sciences, James Hutton Institute, Invergowrie, Dundee, DD2 5DA Scotland; 4 School of Engineering, The University of Glasgow, Oakfield Avenue, Glasgow, G128LT UK

**Keywords:** Anaerobic digestion, Metagenomics, Sanitisation, Slurry co-digestion

## Abstract

**Background:**

The urgent need to adopt sustainable agricultural practices has positioned anaerobic digestion (AD) as a pivotal technology. Indeed, slurry-based AD can mitigate agricultural pollution by capturing greenhouse gas from stored slurry and converting it into biomethane, a valuable source of renewable energy, while generating digestate that can be used as fertiliser. For such a strategy to be effectively and widely deployed however, AD must be optimised. To this end, efforts have typically focused solely on biogas yields, yet improvements in pathogen load reduction may potentially negate the need for a costly pasteurisation step. Hence, optimisation of AD for sanitisation as well as improved biogas output is desirable. To address this, we set up triplicate 10-L CSTR bioreactors, which were fed with a combination of slurry and fats, oils and grease for 216 days. An organic loading rate (OLR) of 2 g VS L^−1^ d^−1^ was used throughout the trial, with a retention time of 21 days. For the first 98 days, bioreactors were fed each weekday (Monday to Friday), with 3 × feedstock on Fridays to maintain the OLR over the weekend. On Day 99 and for the remainder of the trial, the feeding regime was changed to every three days, still maintaining the 2 g VS L^−1^ d^−1^ OLR. The change in feeding regime was prompted by a noticeable increase in *E. coli* removal on Mondays, indicating that feeding regime could potentially function as a controllable ecological selection pressure.

**Results:**

After an initial period of adaptation to the new operating conditions (from day 99–150), the change in feeding regime resulted in improved *E. coli* removal, achieving consistently the required reduction in numbers to satisfy EU sanitisation standards (< 1000 CFU g^−1^). Additionally, methane production increased significantly in all bioreactors with an average of 58% higher methane yield per gram VS fed when compared to the previous 5-day feeding regime. Interestingly, process optimisation led to a more tailored microbial community as revealed by metagenomics. Specifically, we observed selection for improved carbon oxidation, syntrophic acetate oxidation and methanogenesis, as well as overall reduced microbial richness and decreased functional diversity. This could potentially lead to a reduced ecosystem stability however the emergence of *Methanosarcina* prevalence, known for its robustness, together with the detection of the two main methanogenic pathways—acetoclastic and hydrogenotrophic—after process optimisation might confer some resistance against future perturbations. The impact of microbial shifts on ecosystem stability needs to be further assessed experimentally.

**Conclusions:**

Taken together, we demonstrate that feeding regime can function as a microbial selection pressure in anaerobic digestion. The switch from a 5-day to a 3-day feeding regime led to shifts in microbial pathways, underpinning the simultaneous improvement in methane production and *E. coli* removal. While further research is required to assess the impact of the observed microbial community dynamics on system stability, our findings suggest that full scale on-farm AD operators could explore the effects of feeding intervals on their process performance.

**Supplementary Information:**

The online version contains supplementary material available at 10.1186/s40793-026-00902-x.

## Background

Global pressure to reduce greenhouse gas (GHG) emissions while maintaining a sustainable level of agricultural production has positioned anaerobic digestion (AD) as a key technology. Indeed, slurry-based AD can reduce agriculture-associated pollution by capturing GHG that would otherwise be emitted from stored slurry and converting it to a usable source of energy, namely biomethane. Thus, slurry-based AD can play a dual role in decarbonising the agriculture sector and the gas grid. Ambitious GHG emission reduction targets have been set in the EU and elsewhere [1] hence promoting biomethane production through AD. To meet these targets, however, optimisation of existing AD systems is urgently required.

Another advantage of slurry-based AD lies in the generation of digestate, which is a nutrient rich biofertiliser. Animal slurry, however, typically contains a range of potential pathogens which pose significant risk to human health [2]. Thus, the removal of these pathogens during the process is an important consideration when seeking to leverage the full potential of agriculture-based AD. Furthermore, due to its low methane yield, slurry is often co-digested with other organic substrates including food processing wastes [3]. While these feedstocks bolster methane production, they can also include animal by-products (ABP) and are consequently associated with an increased disease transmission risk to humans and animals. Although a pasteurisation step is typically required for AD plants processing ABP it is not always mandated, for example, when using grease trap waste as a co-digestion feedstock [4]. These substrates are commonly used for co-digestion as they are readily available since food processing facilities and restaurants are required to prevent escape of fats, oils and grease (FOG) to wastewater systems [5].

Typically, pathogen load in digestate is monitored using faecal indicators, such as *Escherichia coli*, as a proxy. Indeed, regular *E. coli* testing is required to ensure satisfactory reduction below 1000 colony forming units (CFU) per gram, in four of five samples, and up to 5000 CFU per gram in one of five samples [4, 6]. Several factors contribute to the removal of *E. coli*, and theoretically by extension, pathogenic agents. Foremost of these factors is temperature, with thermophilic AD consistently demonstrating superior *E. coli* removal compared with mesophilic or ambient temperature AD [7–14]. Thermophilic plants are, however, typically associated with operational difficulties linked with the inherent instability of highly specialised thermophilic microbial communities [15, 16]. Retention time has also been shown to impact *E. coli* survival [2, 7, 17–19] but beyond twenty days this effect is minimal [9, 20] and most agriculture-based AD plants operate with retention times in excess of fifty days [21]. Organic loading rate (OLR) can also influence pathogen survival, given that theoretically higher concentrations of pathogenic material may be added under higher OLRs [22]. Conversely, higher OLRs can potentially increase concentrations of volatile fatty acids and free ammonia, both of which are associated with pathogen reduction [23–25], particularly at acidic pH [18, 26]. Other factors affecting pathogen survival include mixing efficacy and bypass flow [9], as well as microbial competition in limited resource conditions [23, 27, 28]. Manser et al. (2015, [29]), using pig slurry in unmixed AD systems reported improved *E. coli* removal and biogas yield when feeding weekly rather than every other day. Similarly, Silva et al*.* (2020; [30]), also digesting pig slurry, observed an increase of 34% in methane yield when changing from daily feeding to every two days and of 37% from daily to every three days. *E. coli* detection was < 100 CFU per gram under all conditions investigated [30]. Sahlström (2003; [18]), however, has highlighted economic and practical reasons for the justification of continuous feeding (typically once per hour, or at least once per day) prevailing in full-scale systems. Nonetheless, as agriculture-based AD technology is maturing, the desire to optimise the system and maximise output has led to some developments in approach to feeding regime [31]. In particular, some scope has emerged for improving functional stability in AD systems generally [32, 33] and more specifically, biogas output from AD of fats, oils and grease [34] via pulse or semi-continuous feeding regime, instead of constant or daily feeding. The possibility of manipulating feeding regime to improve AD of FOG has been demonstrated, but the effect on biogas output from co-digestion of slurry with FOG remains unclear [34]. Furthermore, the associated potential for improved sanitisation via feeding manipulation is unknown.

As AD is a microbial-driven process, any optimisation strategy needs to investigate the impact of changing operational parameters on microbial dynamics. Typically, 16S rRNA sequencing is used to characterise AD microbiomes [35], but this provides limited information on the potential functioning of ecosystems. This can be examined, however, with metagenomics, which can link taxonomy to potential functions, thereby providing valuable insights into ecosystem functioning, including functional redundancy, inherently linked to process stability. Thus, the overarching aims of this work were to i) optimise the co-digestion of slurry and FOG in terms of biomethane yield and sanitisation using feeding regime as a controllable microbial selection pressure and ii) investigate the corresponding microbial dynamics underpinning the observed AD microbiome phenotypes using metagenomics.

## Methods

### Feedstock and inoculum

FOG were sourced and collected once in a 125-L drum from an AD plant in County Kilkenny, Ireland, while dairy cattle slurry (DCS) was collected from a dairy farm in County Galway, Ireland. The underground slatted storage tanks were mechanically agitated to homogenise the slurry before collection using a bucket attached to a pole [36]. Both FOG and DCS were stored at 4 °C prior to use. To prepare the AD feedstock, DCS was mixed with FOG at a 2:1 DCS:FOG ratio, based on common practice at the source farm-based AD plant (County Kilkenny). Seed inoculum was obtained from a full-scale AD plant feeding slurry and FOG in Co. Kilkenny, Ireland. Triplicate 10L laboratory-scale bioreactors (8L working volume) were operated identically for 240 days. After 67 days of operation, slurry was collected weekly, to avoid reduction of *E. coli* load during storage. The mixed feedstock was monitored at each time point for TS, VS, pH, tCOD, sCOD, NH_3_ and *E. coli* before being fed through the feeding port on top of each bioreactor.

### Feeding regime

An organic loading rate (OLR) of 2 g VS L^−1^ d^−1^ was used throughout the trial, with a retention time of 21 days. Feedstock VS content was determined every two weeks to account for any fluctuations in solids between DCS collections. For the first 98 days, bioreactors were fed and sampled each weekday (Monday to Friday), with 3 × feedstock each Friday to maintain the 2 g VS L^−1^ d^−1^ OLR over the weekend—this period is designated Phase 1. On Day 99, the feeding regime was changed to every three days, still maintaining the 2 g VS L^−1^ d^−1^ OLR. Hence, in a 21-day cycle, bioreactors received an average of 42 g VS L^−1^ (8-L working volume), regardless of the feeding regime employed. The three-day semi-continuous feeding regime was maintained until the end of the trial—this covers Phase 2 (the acclimatisation phase; Day 99–Day 150) and Phase 3 (Day 150–Day 216). The equivalent volume of digestate to that being fed was removed from each digester prior to feeding, throughout the reactor trial.

### Physicochemical analysis

Biogas produced by the bioreactors was collected in 25-L Tedlar SCV gas bags. The biogas volume was determined at each sampling point using the water displacement method. Methane content of the biogas was analysed using a Varian 450 gas chromatograph (GC) equipped with a flame ionisation detector. The carrier gas was nitrogen, and the flow rate was 25 mL min^−1^. Analysis of TS and VS was performed gravimetrically, while tCOD and sCOD analysis was performed via titration, as described in the standards methods [37]. NH_3_ concentrations (mg L^−1^) were determined using the HACH AmVer High-Range Ammonia test, following the manufacturer’s instructions. TS, VS, pH, NH_3_ and sCOD data are available in Supplementary Materials (Additional file 1 and Additional file 2).

### *Escherichia coli* monitoring

Samples were taken prior to each feeding event for the determination of *E. coli* most probable number (MPN), using IDEXX Colisure with Quanti-Tray/2000 incubated at 35 °C for 24 h. *E. coli* numbers were recorded in influent and effluent using serial dilutions as concentrations were substantially higher in the influent. The test kit has a 0–2419.6 MPN/100 ml range. *E. coli* removal was calculated as the difference between influent and effluent numbers collected at the next timepoint. FOG samples were analysed fortnightly and were found to consistently have *E. coli* numbers below the limit of detection (100 CFU g^−1^).

### DNA extraction

At each feeding event, 2 mL aliquots were sampled from each bioreactor. These were flash frozen in liquid nitrogen and stored at − 80 °C. Triplicate samples (one from each replicate reactor) from eight time points were selected for metagenomic analysis (n = 24). During Phase 1, four sampling points were chosen before and after weekend feeding (Thursdays and Mondays; D78/D82 and D92/D96). During Phase 2, the acclimatisation phase, two sampling points were selected, one at the beginning and one at the end (D102 and D123). Finally, during the stable three-day semi-continuous feeding phase (Phase 3) two sampling points were chosen, one at the beginning and one at the end (D153 and D213). DNA was extracted from the 24 samples using the method developed by Thorn et al. (2019; [38]). Extracts were stored at − 80 °C prior to metagenomic sequencing.

### Metagenomic sequencing, assembly and annotation

Shotgun sequencing was carried out by Teagasc Sequencing Platform at Moorepark (Ireland), with two runs of 12 samples each in Illumina NextSeq High Output 300 cycles. The raw sequence files are available in the European Nucleotide Archive under the project accession number PRJEB85511 with sample details provided in Additional file 3. A total of 24 metagenomics samples were processed. Adapter trimmed reads were provided by the sequencing centre. These were subjected to quality trimming using Sickle v1.200 [39] with an average Phred quality below 20, while retaining paired-end reads greater than 50 bp. This resulted in a total of 820,722,336 reads from all samples. Then all forward and reverse reads were used for an all-samples co-assembly using megahit with the parameters—k-list 27,47,67,87 k min^−1^pass -m 0.95—min-contig-len 1000 [40]. This led to a total of 531,216 contigs, comprising 1,457,849,774 bp with maximum contig size of 318,405 bp, average contig size of 2744 bp, and N50 score of 3445 bp. The resulting contigs were binned using the MetaWRAP pipeline [41]) with three different binning algorithms: metabat2 (443 bins), maxbin2 (520 bins), and CONCOCT (377 bins). checkM [42] was then applied to assess bin completion as well as contamination (including checking for chimera). Bins from the three binning algorithms were consolidated, within the MetaWRAP framework, to only retain bins with 50% completion, and 10% contamination, which led to a final set of 358 bins or metagenomic assembled genomes (MAGs). A mean genome completion of 82.20% was obtained, with a mean contamination of 1.843% as determined using CoverM [43]. Then, we used the METABOLIC pipeline [44] to analyse metabolic functions encoded in MAGs and investigate nutrient cycling (carbon, nitrogen, sulfur, including taxonomy using GTDB-TK; [45]). METABOLIC recovers annotation of proteins using KEGG [46], TIGRfam [47], Pfam [48], custom hidden Markov model (HMM) databases [49], dbCAN2 [50], and MEROPS [51]. The obtained sample read coverages per bin *C*_*i,j*_ was multiplied with feature coverages (returned from METABOLIC) per bin *F*_*j,k*_ to obtain feature coverages per sample *n*_*i,k*_ as a matrix product *n*_*i,k*_ = _*j*_* C*_*i,j*_* F*_*j,k*_. Only high-quality bins were used with minimum genome completeness of 75% and maximum genome contamination of 5%, which resulted in a final table of n = 24 × p = 254 bins table. From this, sample-wise abundance tables could be derived: dbCan2 (n = 24 samples × *p* = 147 features, i.e., CAZymes); Function Traits (n = 24 samples × *p* = 82 features); HMM Hits (n = 24 samples × *p* = 190 features); KEGG Modules (n = 24 samples × *p* = 353 features); KEGG Submodules (n = 24 samples × *p* = 1240 features); and MEROPS (n = 24 × *p* = 142 features). Next, GToTree [52] was used to explore phylogenetic relationships between MAGs. This algorithm detects Single Copy Genes (SCGs) in MAGs and then multisequence aligns them to generate a phylogenetic tree. For this purpose, the reference HMM SCGs set available in the software was used as follows: i) 74 bacteria specific SCG markers; ii) 76 archaea specific SCG markers; and iii) universal 16 SCG markers [53]. Bins that had very few hits to SCGs were removed, and the final phylogenetic tree contained: 276 bins for bacteria specific SCG markers, 11 bins for archaea specific SCG markers, and 275 bins for universal SCG markers, when only bins with a minimum of 75% completion and a maximum of 5% contamination were considered.

### Statistical analysis

A Generalised Linear Latent Variable Model (GLLVM) was used to investigate the relationship between microbial genera and environmental conditions prevailing during the three experimental phases. GLLVM extends the basic generalised linear model that regresses the mean abundances ${\mu }_{ij}$ (for $i$-th sample and $j$-th MAG) against the covariates ${x}_{i}$ by incorporating latent variables ${u}_{i}$ as $g\left({\mu }_{ij}\right)={\eta }_{ij}={\alpha }_{i}+{\beta }_{0j}+{x}_{i}^{T}{\beta }_{j}+{u}_{i}^{T}{\theta }_{j}$, where ${\beta }_{j}$ are the MAG specific coefficients associated with individual covariate and ${\theta }_{j}$ are the corresponding coefficients associated with latent variable. ${\beta }_{0j}$ are the MAG-specific intercepts, whilst ${\alpha }_{i}$ are optional sample effects which can either be chosen as fixed effects or random effects. A 95% confidence interval of ${\beta }_{j}$ coefficients whether positive or negative, and not crossing 0 boundary can be interpreted as an increase or decrease in that particular covariate, which reflects an increase or decrease in the abundance of the MAG. For convergence of GLLVM algorithm, family = poisson() and method = “LA” in the gllvm() function were used. Finally, MAG novelty was examined using the Genome Tree Toolkit (https://github.com/donovan-h-parks/GenomeTreeTk) by checking phylogenetic gain for each MAG against the rest of the tree, with higher values potentially identifying novel species. These were calculated for each MAG in the trees recovered for bacteria, archaea and universal SCGs, respectively.

## Results

### Feeding regime impacts methane production and *E. coli* removal

**Fig. 1:**
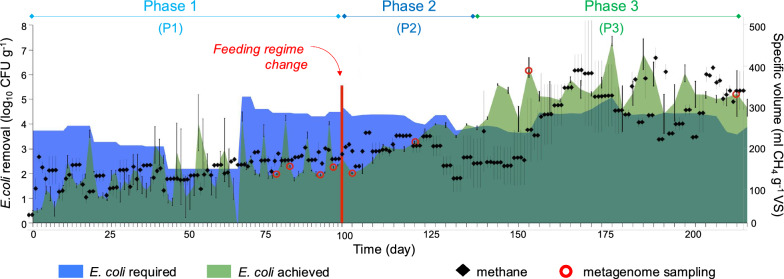
*E. coli* removal and biogas production with light green shading denoting the achieved *E. coli* removal concentrations and blue denoting the required *E. coli* removal concentrations to satisfy the EU digestate standard of < 1000 CFU g^−1^. Dark green shading corresponds to an overlap between light green (achieved *E. coli* removal) and blue (required *E. coli* removal). Black diamonds indicate methane production with standard error bars (*n* = 3). The red line indicates change of feeding regime at Day 99. Red circles indicate sample points for metagenomics

During Phase 1 of reactor operation and regardless of initial levels in the feedstock, *E. coli* removal appeared to peak at regular intervals, which corresponded to Monday’s samples (Fig. 1; light and dark green shading). It is worth noting that DCS was stored at 4 °C for the first 66 days of the trial, which resulted in a reduction in initial *E. coli* levels in the feedstock (Fig. 1; blue shading). Beginning on Day 67, fresh DCS was collected every two weeks. Following a transition phase, after the feeding regime change, Phase 2 (from day 99 to day 150) *E. coli* removal steadily improved until the EU digestate standard (< 1000 CFU g^−1^) was consistently achieved beyond Day 150 to the end of the trial (Fig. 1; light and dark green shading). Remarkably, concomitant with improved *E. coli* removal, increased biomethane production was observed as a result of the changed feeding regime. *E. coli* reduction slightly pre-empted the altered trend observed for methane production (Fig. 1). During Phase 2, directly following the feeding regime change, methane production fell from a stable average during Phase 1 of 249 ± 5 mL CH_4_ g VS^−1^ to 119 ± 32 mL CH_4_ g VS^−1^ between Day 130–133 (Fig. 1). In Phase 3, methane production increased from Day 150 to a peak of 690 ± 7.4 mL CH_4_ g VS^−1^ fed as excess COD was consumed, before stabilising from Day 180 until the end of the trial at an average of 430 ± 7.4 mL CH_4_ g VS^−1^ (Fig. 1).

### Change in feeding regime induces microbial community shifts

**Fig. 2:**
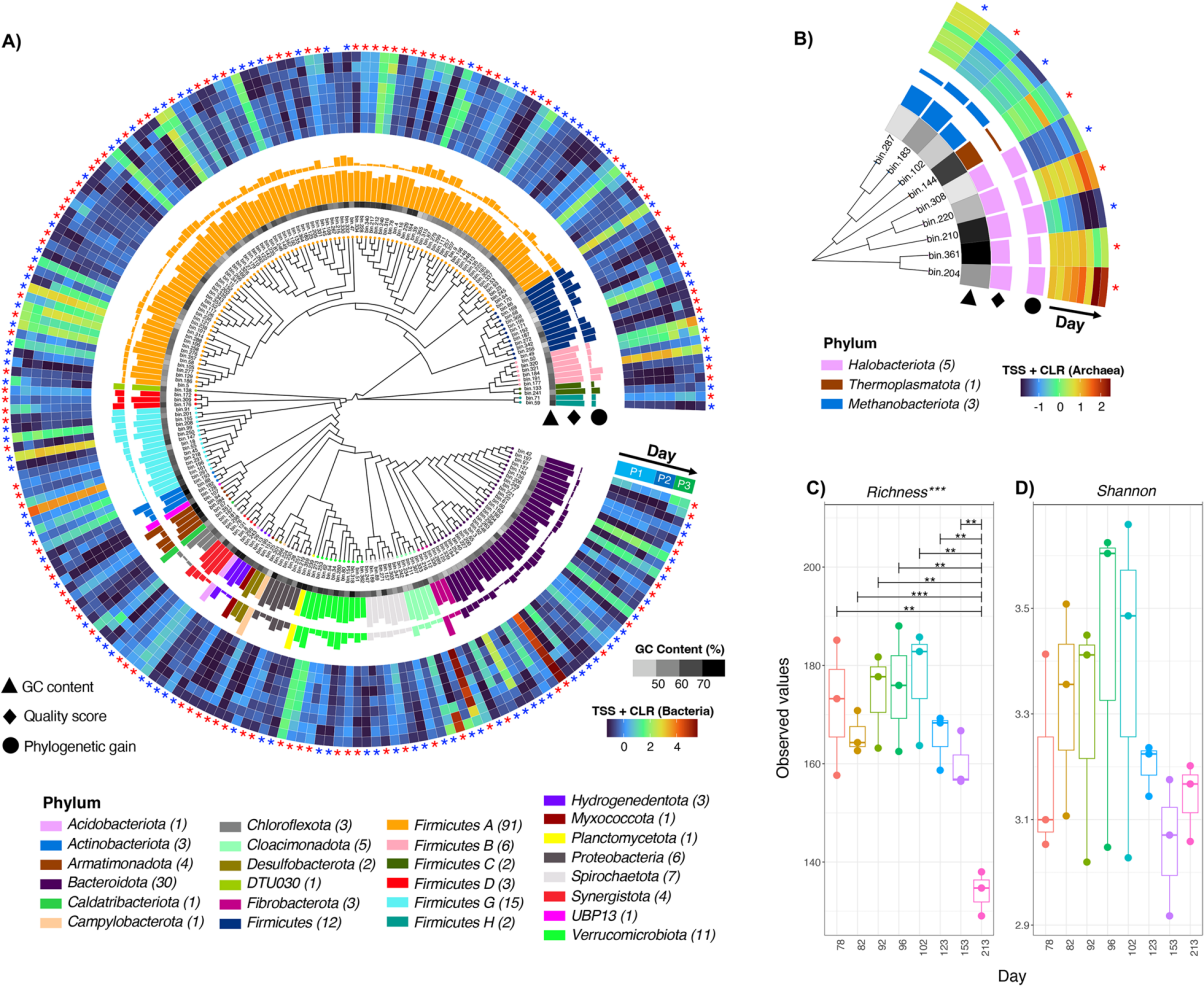
Diversity and abundance of MAGs showing **A** the phylogenetic tree recovered by GToTree using 74 bacterial specific single copy genes (SCGs) and, **B** the archaeal tree generated using 76 archaeal SCG markers. Figures **A** and **B** also show GC content, quality score (genome completion—5 × genome contamination), phylogenetic gain (PG) and sample abundances (heatmap; TSS + CLR) for each of the recovered MAGs. Red and blue asterisks represent the positive and negative beta coefficients, respectively, for each genome, returned by GLLVM against changes in feeding regime. Alpha diversity is represented by rarefied richness **(C)** and Shannon entropy **(D)**, coloured according to day of sampling collection

Metagenomic sequencing of 24 biomass samples yielded a total of 820,722,336 reads. Assembly and binning resulted in 358 MAGs with a minimum of 50% completion, and a maximum of 10% contamination. The 358 MAGs comprised 20 different phyla, the majority identified as *Firmicutes* (Fig. 2A). Nine methanogenic archaea were identified and belonged to the phyla *Halobacterota*, *Thermoplasmatota*, and *Methanobacteriota* (Fig. 2B). Changes in the feeding regime resulted in increased abundances of 5 of these methanogens. Notably, *Methanothrix* (Bin.210) decreased in abundance following the feeding regime change, while its competitor for acetate, *Methanosarcina* (Bin.220) increased in abundance (Fig. 2B; Additional file 4). Most bacterial phyla contained a mix of bins that either increased or decreased without strong patterns. Minimal changes were observed in terms of alpha diversity (Fig. 2C and D), however gradients and clustering could be seen for beta diversity (Additional file 5). The changes in community structure correlated with both reduced *E. coli* numbers, and with increased methane production (Additional file 5). Calculating the phylogenetic gain (PG) of each bin identified those that were potentially novel. The 10 bins with the highest PG came from 8 different phyla, 3 of these bins belonging to the phylum *Patescibacteria* (Additional file 6). It is worth noting that we could not detect *E. coli* in our metagenomic dataset.

### Change in feeding regime affects potential microbiome function

**Fig. 3:**
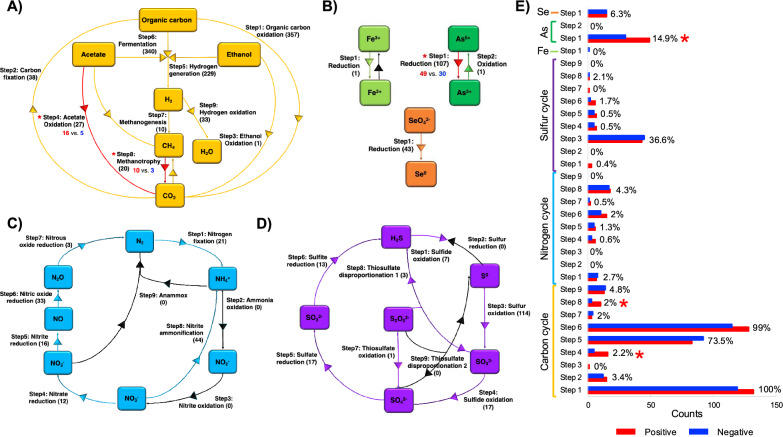
Major geochemical cycles **(A–D)** returned from METABOLIC with the count showing the number of MAGs that have that particular pathway, along with the coverage information for all MAGs recovered in this dataset (displayed as percentage in panel **E**). Additionally, associations to feeding regime changes are indicated showing the number of genomes with positive association to feeding regime changes (red), and the number of genomes with negative associations to feeding regime changes (blue). Red arrows **(A, B)** and asterisks **(E)** represent those pathways that were substantially more abundant in the community after the feeding regime change. These associations are based on GLLVM analysis where coefficient ${\beta }_{j}$ and its 95% confidence interval were used to determine the direction and significance of the effect. A coefficient was considered positive if ${\beta }_{j}$> 0 and the 95% internal lay entirely above zero and negative if ${\beta }_{j}$< 0 and the 95% internal lay entirely below zero. A black arrow **(A–D)** indicates that this pathway was not detected in any of the recovered MAGs

The microbial community was found to contain all major steps involved with carbon transformations (Fig. 3A). All the bins (357; 100% coverage) contained pathways involved in organic carbon oxidation (Step 1; Fig. 3A). Changing the feeding regime resulted in a community structure with more bins capable of this step (132) than bins that were not (119). Similar patterns were observed for Steps 1, 2, 3, 4, 6, and 8 of the carbon cycle (Fig. 3A). We can infer that Step 7 (methanogenesis; Fig. 3A), was also potentially upregulated due to the increase in abundance of methanogenic archaea (Fig. 2B), but it was not captured by this particular analysis (given that two of the archaeal MAGs were not represented). Acetate oxidation (Step 4) and methanotrophy (Step 8) were particularly positively influenced by the feeding regime changes, appearing to funnel carbon towards CO_2_ (Fig. 3A). Complete nitrogen and sulfur cycles could not be identified within the MAGs of the microbial community (Fig. 3C and D). There was also no substantial difference between the number of MAGs containing these steps and their associations with the feeding regime changes (Fig. 3C, D and E). Interestingly, however, more MAGs containing the genes involved in arsenate reduction were positively associated with the feeding regime (Fig. 3B).

**Fig. 4:**
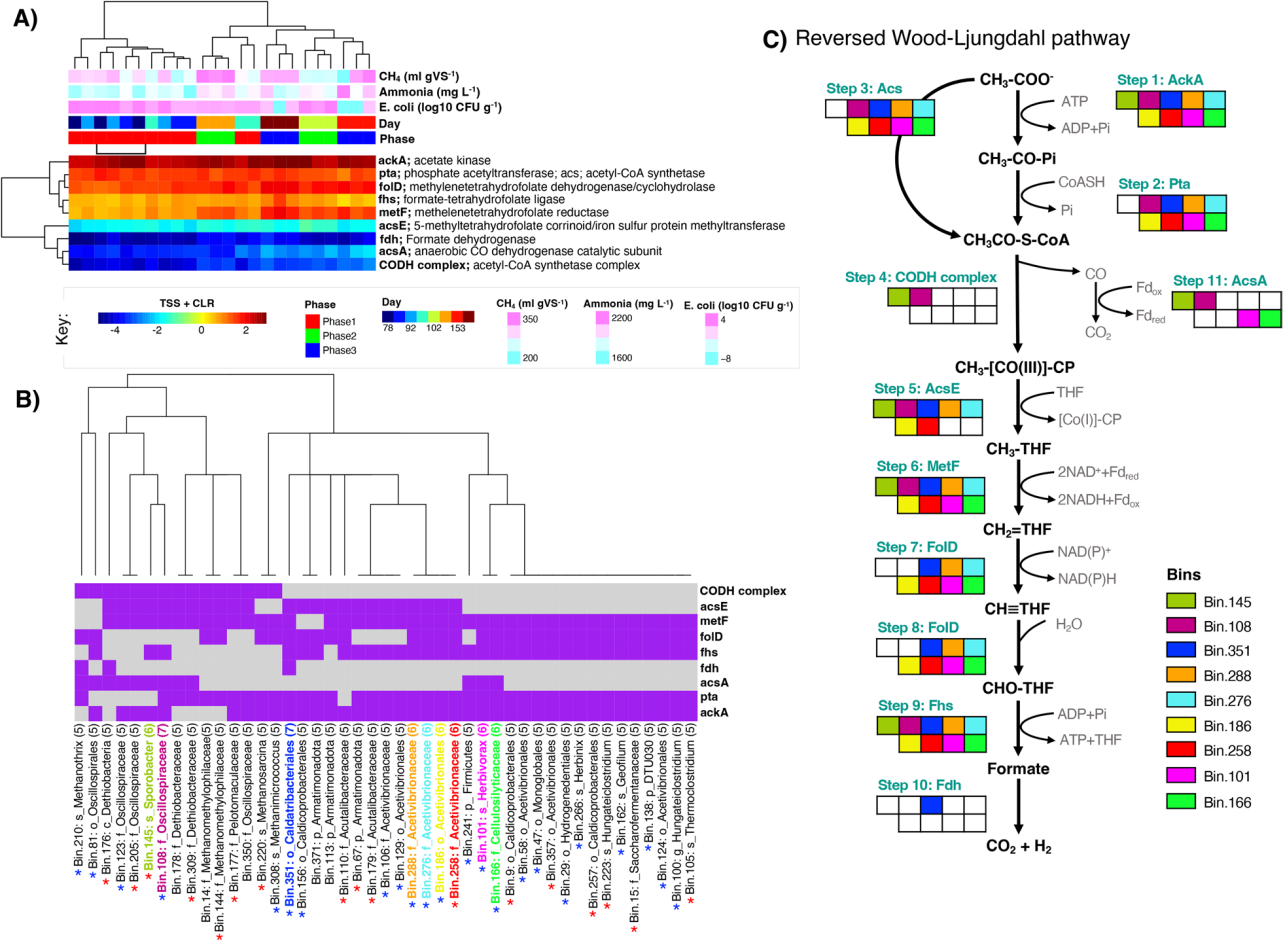
Abundance heatmap **(A)** of KEGG modules associated with the reversed Wood-Ljungdahl (WL) pathway for syntrophic acetate oxidising bacteria (SAOB) metabolism annotated (above) with sources of variability. Rows and columns are organised according to hierarchical clustering using average linkages. Presence/absence heatmap **(B)** of the taxa containing at least 5 of the same modules identified in **(A)**, those taxa containing at least 6 of the 9 modules comprising the WL pathway were coloured and used to create the pathway map **(C)**. Red and blue stars in **(B)** indicate those bins whose abundances are positively (red) or negatively (blue) associated with the feeding regime change

Given the increased abundance of genes related to syntrophic acetate oxidation and methanogenesis following the feeding regime change (Fig. 2B and Fig. 3A), as well as the importance of these pathways to AD, we decided to target the KEGG modules associated with each of these processes. There are 9 KEGG modules associated with the reversed Wood-Ljungdahl (WL) pathway, the only confirmed pathway available to syntrophic acetate oxidising bacteria (SAOB). All 9 of these modules were identified within the microbial community. Indeed, several of these modules were highly abundant throughout the trial (Fig. 4A). Moreover, some of these modules became more abundant during Phase 3 (after the feeding regime change) as predicted by the METABOLIC carbon cycle diagrams (Fig. 3A)—these encode for enzymes: AckA (acetate kinase), MetF (methylenetetrahydrofolate dehydrogenase/cyclohydrolase) and AcsA (anaerobic CO dehydrogenase catalytic subunit). None of the recovered bins were found to contain all 9 of the modules associated with the WL pathway, however 45 bins were identified which contained at least 5 of the WL modules (Fig. 4B). Of these, only 9 bins contained 6 or more of the modules (Fig. 4B and C).

**Fig. 5:**
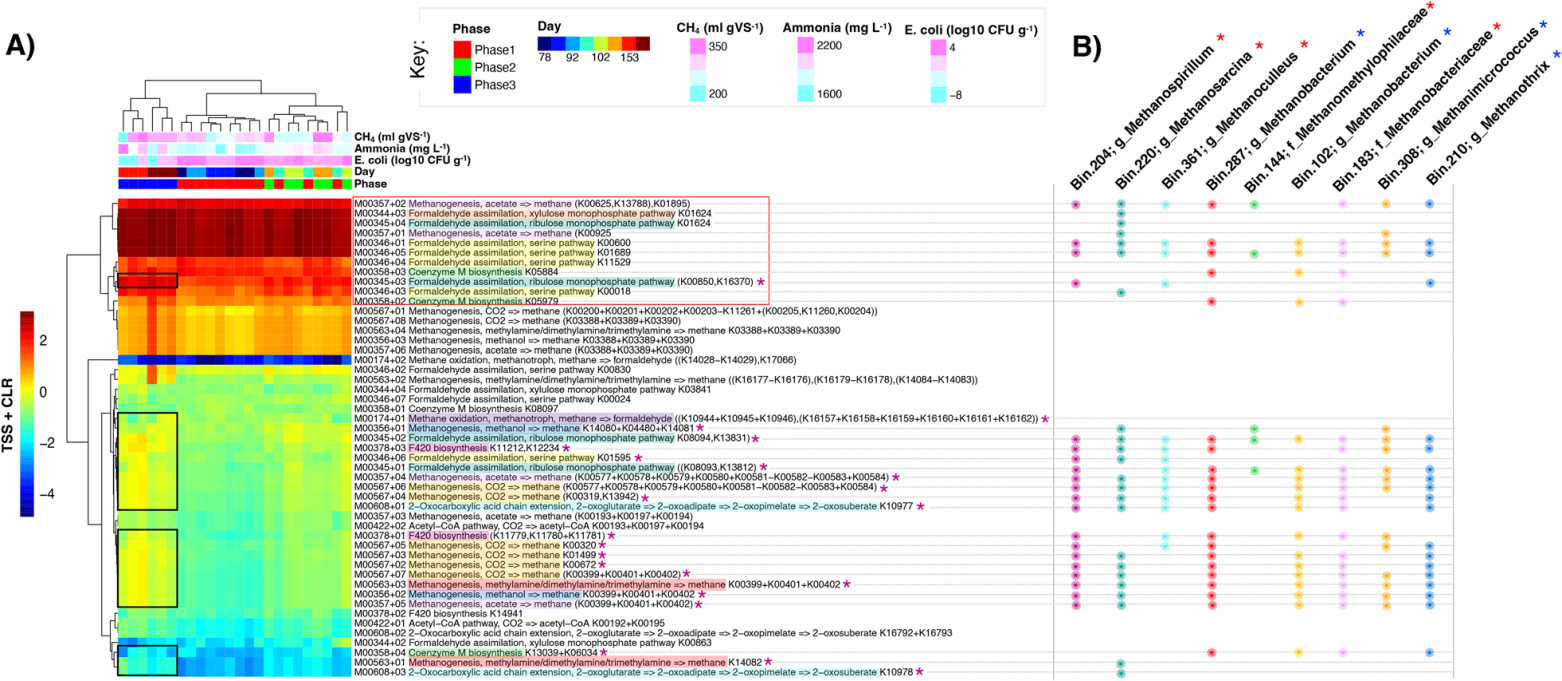
Heatmap **(A)** of KEGG modules associated with methane metabolism returned by METABOLIC and annotated (above) with sources of variability. Rows and columns are organized according to hierarchical clustering using average linkages. Pathways which were upregulated during Phase 3 (after the feeding regime change) are highlighted in the heatmap with a black box and a pink star. Those pathways which were abundant throughout all the phases are highlighted in the red box. Pathways which were more abundant in Phase 3 and/or abundant throughout are coloured for readability. The dot plot **(B)** shows the methanogenic MAGs, with associated KEGG pathways. Asterisks indicate those positively (red) and negatively (blue) associated with the feeding regime change

Finally, the KEGG modules associated with methane metabolism present within the microbial community were identified (Fig. 5A). Many of these modules increased in abundance during Phase 3 (following the feeding regime change). These included pathways coding for methanogenesis (autotrophic and heterotrophic), formaldehyde assimilation, F20 biosynthesis and coenzyme M biosynthesis. Most of the methanogenic archaea contained many of these modules, however, bin.144 (81.31% complete), identified as a member of the family *Methanomethyolphilaceae*, only contained 5 modules pertaining to methane metabolism—substantially fewer than the other archaea (Fig. 5B).

## Discussion

Here, we demonstrated that feeding regime can function as a microbial selection pressure in farm-based anaerobic digestion. Specifically, we report that switching from a 5-day to a 3-day feeding regime led to a pathway-level shift in methanogenesis, underpinning the simultaneous improvement in methane production and pathogen indicator removal. Addressing multiple aspects of the AD process was previously highlighted for adequate system optimisation [54]. In this context, substrate feeding rate was identified as a key parameter to define optimal operating conditions, with the multiple objectives of maximising biogas yields, profits and saving greenhouse gas emissions. Thus, our results extend this observation to the optimisation of both biogas production and digestate sanitisation, confirming that feeding regime can be used as a controllable microbial selection pressure. Other approaches to agriculture-based AD have recently involved two-stage systems, including a mesophilic and a thermophilic step, where cow manure was co-digested with yeast extract [55]. This strategy led to digestate sanitisation and the production of 421 mL CH_4_ g VS^−1^, which is on par with our results.

### Process optimisation yielded a more tailored microbial community

During Phase 1, microbial community richness assessed from DNA did not seem to be impacted by the Friday increased feedstock, even though phenotypically, *E. coli* removal improved as a result. Upon switching to three-day feeding and hence repeating the Friday feeding patterns, within 20 days of operation, microbial community richness seemed to decrease and became significantly lower by the end of the trial. This may indicate that the optimisation process led to the emergence of a tailored community. It is worth noting that this decreased richness also translated into reduced functional diversity on the final sampling day (Additional file 7). This, in turn could make the system less stable, and more sensitive to disturbances [56, 57]. However, the balance between positive and negative interactions between microorganisms within ecosystems has been suggested to underpin system stability [58], while community resilience has been linked to the prevalence of large populations of generalist species and not diversity per se [59]. Hence, the impact of our observed reduced microbial community richness and associated decreased functional diversity would be best tested experimentally by subjecting two sets of replicate reactors under stable operating conditions, each with one of our two feeding regimes, to further disturbances. The tailored community emerging from our process optimisation consisted of a wide range of bacteria including bins belonging to *Cloacimonadaceae* (Bin.211), *Bacteroidales* (Bin.227), *Limnochordia* (Bin.218), *Fermentimonas* (Bin.42) and *Anaerolineaceae* (Bin.167), among others (Additional file 8). There were also interesting shifts among the methanogenic archaea with *Methanothrix* (Bin.210), an acetoclastic methanogen, decreasing significantly following the feeding regime change. Meanwhile, *Methanospirillum* (Bin.204) and *Methanosarcina* (Bin.220) increased, representing dominance of hydrogenotrophic methanogenesis. *Methanosarcina*, in particular has been shown to act as an important and resilient buffer against perturbations [60, 61]. Indeed, *Methanosarcina* can use acetate, hydrogen/CO₂, and methylated compounds as substrate, conferring these methanogens a somewhat unique flexibility underpinning their survival and contribution to methane production during changing environmental conditions [60]. Furthermore, they are tolerant to high ammonia concentrations (up to 7000 mg L^−1^), high salt concentrations (up to 18,000 mg Na^+^ L^−1^), high acetate concentrations (up to 15,000 mg COD L^−1^) as well as pH shock (0.8–1.0 units) [60]. *Methanosarcina*, has also been associated with increased bioreactor performance during grass and sewage sludge co-digestion [62]. Despite the observed reduced microbial diversity after process optimisation, the prevalence of *Methanosarcina* in our tailored community, together with detection of the two dominant methanogenic pathways, might confer some resistance against ecosystem perturbation. It is worth noting that we could not detect *E. coli* in our metagenomic datasets. However, we detected a maximum of 10^5^ CFU/g at the time of sampling for metagenomics (on day 78, 92 and 102; data not shown), likely representing < 0.1% of the microbial community, which corresponds to the threshold of detection for the Illumina platform used herein. This highlights the importance of combining molecular and culture-based approaches when assessing the potential risk of digestate. It is also important to note that we only focused on *E. coli*, while other pathogen indicators representative of Gram-positive (e.g. enterococci), spore formers (e.g. *Clostridium sp*), or yeast (e.g. *Candida albicans*) would have provided relevant information on more resistant pathogens. Finally, taxonomic abundance and metabolic functions were found to be significantly different when using *E. coli* as a covariate (Additional File 9). This suggests that pathogen indicators, and perhaps pathogens, might influence microbial community structure and functioning. To clarify this, future research could assess the effects of pathogens on farm-AD microbiomes and associated process performance.

### Optimisation resulted in a shift in methanogenic pathways

The microbial community responded significantly to the changing operational conditions. Strong correlations were observed between the different phases and the microbial community composition (*p* = 0.0001; Additional file 9). Of the 9 methanogenic archaea identified, 5 of these were positively correlated with the change in feeding regime according to GLLVM, and three of these (Bin.220, Bin.361 and Bin.204) were present in high abundances throughout the study. An increase in methanogenesis was not however, represented by the nutrient cycling diagrams generated by METABOLIC. Indeed, this analysis suggested an increase in methanotrophy, rather than methanogenesis. While genes for methanotrophy were detected within the community, the taxa capable of methanotrophy were present only in very low abundances and there was no apparent loss of CH_4_ to this pathway. Analysis of all the KEGG modules involved in methane metabolism revealed substantial capability for formaldehyde-driven methanogenesis as well as an increase in Phase 3 for autotrophic methanogenesis from CO_2_ and H_2_. This presented as a shift from acetate-driven methanogenesis towards acetate oxidation (by SAOBs) and subsequent methanation of CO_2_, likely resulting in an overall increase in methane production. Such shift has been previously reported in anaerobic digestion, particularly in the context of high ammonia concentrations [63–65], which is in line with our observed increased ammonia concentrations prevailing after process optimisation. Indeed, acetotrophic methanogens have been reported to be inhibited at elevated ammonia concentrations hence opening a niche for syntrophic acetate oxidation (SAO) coupled with hydrogenotrophic methanogenesis [63–65]. Interestingly, in CSTR digesting synthetic raw domestic sewage the switch from daily to every two-day feeding has been reported to promote tolerance to organic shock load and high ammonia concentrations [32]. Taken together, here we demonstrate a link between process conditions and amplification of particular metabolic pathways.

## Conclusions

We report the simultaneous optimisation of methane production and *E. coli* removal during the co-digestion of slurry with FOG using feeding regime as a controllable microbial selection pressure. This provides a solid starting point for full scale on-farm AD, whereby operators can investigate the impact of feeding regime on their process performance. Full-scale AD plants typically have more than one primary digester, often as many as four. In such a scenario the pulse feed could be alternated/rotated so that gas volume and quality remain steady and the same volume of feed can be stored. As most commercial AD plants do not intake feed continuously, there are natural ebbs and flows in storage, hence pulse feeding regime would not require any specific scheduling. We noted a tailoring of the microbial community in response to change in feeding regime with a shift from *Methanotrix* to *Methanorcina* as well as an increased capacity for syntrophic acetate oxidation coupled with hydrogenotrophic methanogenesis under optimised conditions. The impact of the reduced richness and functional diversity resulting from the microbial selection pressure described herein on process stability is unclear and required further experimental investigations. Process optimisation typically focuses solely on higher yields however system stability should also be considered. Striving for increased biogas or target compound production from mixed microbial fermentations likely result in a trade-off between microbial diversity and process performance, driven by the selection pressure imposed on the consortium. Such a strategy might suit microbial processes converting homogenous feedstocks into added-value products but is likely not optimal when dealing with waste streams inherently heterogeneous. Future work on process optimisation in this context should therefore aim to address the impact of increased yields on system stability and develop a framework to predict process adaptive capacity to maximise not process performance per se but the trade-off between performance and stability. Finally, it is important to note that metagenomics only provided information on microbial potential capacity and even though the longitudinal nature of our sampling strategy allows for insight into ecosystem functioning further post-genomic analyses are required to verify our observations.

## Supplementary Information

Additional file 1: Physicochemical analysis of samples from triplicate CSTRs processing DCS and FOG for 216 days initially fed daily (first 99 days) and then fed every three days, including pH (A), volatile solids concentration (%; B), NH3 (C) and sCOD (D), with error bars indicating standard error of technical replicates (n = 3). A vertical red line indicates the change of feeding regime on Day 99.

Additional file 2: Average physicochemical data of mixed feedstock throughout trial.

Additional file 3: Metagenomics samples information.

Additional file 4: Microbial community structure shown as the relative abundance (proportions) of the archaeal fraction of the community.

Additional file 5: E. coli removal and methane production. E. coli values were fit on the first two dimensions of the ordination plot using smooth cubic splines (CS) interpolation after fitting generalised additive model of the form E. coli ~ CS (Dim1, Dim2), which returned as significant with p<0.05; phases are represented by shape, size of the shape represents the quantity of E. coli and the heatmap shows the quantity of methane generated at those timepoints.

Additional file 6: Novel bins. Phylogenetic gain (PG) was calculated using GTDBTK toolkit with higher values representing novelty of a particular genome within the context of the phylogenetic tree; the 10 most novel bins are shown.

Additional file 7: Functional microbial community diversity measured as (A) richness and (B) Shannon entropy of the recovered KEGG Modules (KEGG Module Hit), coloured according to day of sample collection.

Additional file 8: Microbial community structure shown as the relative abundances (proportions) of the Top-25 most abundant MAGS.

Additional file 9: PERMANOVA results for key environmental covariates where R^2^ explains percentage variability (scaled to 1) when significant (*p* ≤ 0.05). Each abundance table is an N samples x P features table mainly from METABOLIC software with the Bray-Curtis distance between samples supplied to the PERMANOVA procedure along with the covariate’s data. Here N.S. represents non-significant results.

## Data Availability

The datasets generated and analysed during the current study are available in the European Nucleotide Archive under the project accession number PRJEB85511 (https:/www.ebi.ac.uk/ena/browser/view/PRJEB85511). All other data are included within the article and its additional files.

## References

[1] IEA, Net zero roadmap: a global pathway to keep the 1.5 °C goal in reach, IEA, Paris https://www.iea.org/reports/net-zero-roadmap-a-global-pathway-to-keep-the-15-0c-goal-in-reach, Licence: CC BY 4.0. 2023.

[2] Nag R, Whyte P, Markey BK, O’Flaherty V, Bolton D, Fenton O, et al. Ranking hazards pertaining to human health concerns from land application of anaerobic digestate. Sci Total Environ. 2020;710:136297. 32050363 10.1016/j.scitotenv.2019.136297 PMC7126561

[3] Ma G, Ndegwa P, Harrison JH, Chen Y. Methane yields during anaerobic co-digestion of animal manure with other feedstocks: a meta-analysis. Sci Total Environ. 2020;728:138224. 32361106 10.1016/j.scitotenv.2020.138224

[4] DAFM. Approval and operation of biogas plants transforming animal by-products and derived products in Ireland: conditions for approval and operation of biogas plants transforming animal by-products and derived products in Ireland. 2014.

[5] Long JH, Aziz TN, de los Reyes FL, Ducoste JJ. Anaerobic co-digestion of fat, oil, and grease (FOG): a review of gas production and process limitations. Process Saf Environ Prot. 2012;90:231–45.

[6] EC. Regulation (EC) No 1069/2009 of the European Parliament and of the Council of 21 October 2009 laying down health rules as regards animal by-products and derived products not intended for human consumption and repealing regulation (EC) No 1774/2002 (Animal by-products Regulation).

[7] Olsen J, Larsen H. Bacterial decimation times in anaerobic digestions of animal slurries. Biol Wastes. 1987;21:153–68.

[8] De Leon C, Jenkins D. Removal of fecal coliforms by thermophilic anaerobic digestion processes. Water Sci Technol. 2002;46:147–52. 12479464

[9] Smith SR, Lang NL, Cheung KHM, Spanoudaki K. Factors controlling pathogen destruction during anaerobic digestion of biowastes. Waste Manag. 2005;25:417–25. 15869985 10.1016/j.wasman.2005.02.010

[10] Avery LM, Anchang KY, Tumwesige V, Strachan N, Goude PJ. Potential for pathogen reduction in anaerobic digestion and biogas generation in Sub-Saharan Africa. Biomass Bioenergy. 2014;70:112–24.

[11] Scaglia B, D’Imporzano G, Garuti G, Negri M, Adani F. Sanitation ability of anaerobic digestion performed at different temperature on sewage sludge. Sci Total Environ. 2014;466–467:888–97. 23973551 10.1016/j.scitotenv.2013.07.114

[12] Moset V, Poulsen M, Wahid R, Højberg O, Møller HB. Mesophilic versus thermophilic anaerobic digestion of cattle manure: methane productivity and microbial ecology. Microb Biotechnol. 2015;8:787–800. 25737010 10.1111/1751-7915.12271 PMC4554467

[13] Nag R, Auer A, Markey BK, Whyte P, Nolan S, O’Flaherty V, et al. Anaerobic digestion of agricultural manure and biomass—critical indicators of risk and knowledge gaps. Sci Total Environ. 2019;690:460–79. 31299578 10.1016/j.scitotenv.2019.06.512

[14] Jiang Y, Xie SH, Dennehy C, Lawlor PG, Hu ZH, Wu GX, et al. Inactivation of pathogens in anaerobic digestion systems for converting biowastes to bioenergy: a review. Renew Sustain Energy Rev. 2020. 10.1016/j.rser.2019.109654.34234614

[15] Hejnfelt A, Angelidaki I. Anaerobic digestion of slaughterhouse by-products. Biomass Bioenergy. 2009;33:1046–54.

[16] Auer A, Vande Burgt NH, Abram F, Barry G, Fenton O, Markey BK, et al. Agricultural anaerobic digestion power plants in Ireland and Germany: policy and practice. J Sci Food Agric. 2017;97:719–23. 27553887 10.1002/jsfa.8005

[17] Kearney TE, Larkin MJ, Frost JP, Levett PN. Survival of pathogenic bacteria during mesophilic anaerobic digestion of animal waste. J Appl Bacteriol. 1993;75:215–9. 8244898 10.1111/j.1365-2672.1993.tb02768.x

[18] Sahlström L. A review of survival of pathogenic bacteria in organic waste used in biogas plants. Bioresour Technol. 2003;87:161–6. 12765355 10.1016/s0960-8524(02)00168-2

[19] Nolan S, Waters NR, Brennan F, Auer A, Fenton O, Richards K, et al. Toward assessing farm-based anaerobic digestate public health risks: comparative investigation with slurry, effect of pasteurization treatments, and use of miniature bioreactors as proxies for pathogen spiking trials. Front Sustain Food Syst. 2018;2:41.

[20] Dennehy C, Lawlor PG, McCabe MS, Cormican P, Sheahan J, Jiang Y, et al. Anaerobic co-digestion of pig manure and food waste; effects on digestate biosafety, dewaterability, and microbial community dynamics. Waste Manag. 2018;71:532–41. 29113838 10.1016/j.wasman.2017.10.047

[21] Campbell J. Technical note TN698 (Revised)/May 2023 anaerobic digestion (AD)–farm scale. Author: Jim Campbell, SAC Consulting. Revised 2023 by Iain Boyd, SAC Consulting. 2023.

[22] Strauch D. Survival of pathogenic micro-organisms and parasites in excreta, manure and sewage sludge. Rev Sci Tech Int Off Epiz. 1991;10:813–46. 10.20506/rst.10.3.565 1782431

[23] Orzi V, Scaglia B, Lonati S, Riva C, Boccasile G, Alborali GL, et al. The role of biological processes in reducing both odor impact and pathogen content during mesophilic anaerobic digestion. Sci Total Environ. 2015;526:116–26. 25925189 10.1016/j.scitotenv.2015.04.038

[24] Dennehy C, Lawlor PG, Croize T, Jiang Y, Morrison L, Gardiner GE, et al. Synergism and effect of high initial volatile fatty acid concentrations during food waste and pig manure anaerobic co-digestion. Waste Manag. 2016;56:173–80. 27389859 10.1016/j.wasman.2016.06.032

[25] Jiang Y, Dennehy C, Lawlor PG, Hu Z, Zhan X, Gardiner GE. Inactivation of enteric indicator bacteria and system stability during dry co-digestion of food waste and pig manure. Sci Total Environ. 2018;612:293–302. 28850849 10.1016/j.scitotenv.2017.08.214

[26] Kunte DP, Yeole TY, Chiplonkar SA, Ranade DR. Inactivation of *Salmonella typhi* by high levels of volatile fatty acids during anaerobic digestion. J Appl Microbiol. 1998;84:138–42. 15244069 10.1046/j.1365-2672.1997.00335.x

[27] Kearney TE, Larkin MJ, Levett PN. Metabolic activity of pathogenic bacteria during semicontinuous anaerobic digestion. Appl Environ Microbiol. 1994;60:3647–52. 7986040 10.1128/aem.60.10.3647-3652.1994 PMC201868

[28] Ward A, Stensel HD, Ferguson JF, Ma G, Hummel S. Preventing growth of pathogens in pasteurized digester solids. Water Environ Res. 1999;71:176–82.

[29] Manser ND, Mihelcic JR, Ergas SJ. Semi-continuous mesophilic anaerobic digester performance under variations in solids retention time and feeding frequency. Bioresour Technol. 2015;190:359–66. 25965953 10.1016/j.biortech.2015.04.111

[30] Silva I, Jorge C, Brito L, Duarte E. A pig slurry feast/famine feeding regime strategy to improve mesophilic anaerobic digestion efficiency and digestate hygienisation. Waste Manag Res. 2021;39(7):947–55. 33280536 10.1177/0734242X20972794

[31] Willeghems G, Buysse J. Changing old habits: the case of feeding patterns in anaerobic digesters. Renew Energy. 2016;92:212–21.

[32] De Vrieze J, Verstraete W, Boon N. Repeated pulse feeding induces functional stability in anaerobic digestion. Microb Biotechnol. 2013;6:414–24. 23302421 10.1111/1751-7915.12025 PMC3917476

[33] Bonk F, Popp D, Weinrich S, Sträuber H, Kleinsteuber S, Harms H, et al. Intermittent fasting for microbes: how discontinuous feeding increases functional stability in anaerobic digestion. Biotechnol Biofuels. 2018;11:274. 30323859 10.1186/s13068-018-1279-5 PMC6173896

[34] Ziels RM, Sousa DZ, Stensel HD, Beck DAC. DNA-SIP based genome-centric metagenomics identifies key long-chain fatty acid-degrading populations in anaerobic digesters with different feeding frequencies. ISME J. 2018;12:112–23. 28895946 10.1038/ismej.2017.143 PMC5737908

[35] Niya B, Yaakoubi K, Beraich FZ, Arouch M, Kadmiri IM. Current status and future developments of assessing microbiome composition and dynamics in anaerobic digestion systems using metagenomic approaches. Heliyon. 2024;10(6):e28221. 38560681 10.1016/j.heliyon.2024.e28221 PMC10979216

[36] Peyton DP, Healy MG, Fleming GTA, Grant J, Wall D, Morrison L, et al. Nutrient, metal and microbial loss in surface runoff following treated sludge and dairy cattle slurry application to an Irish grassland soil. Sci Total Environ. 2016;541:218–29. 26410697 10.1016/j.scitotenv.2015.09.053

[37] APHA. Standard methods for the examination of water and wastewater/prepared and published jointly by American Public Health Association, American Water Works Association and Water Environment Federation. -Version details-Trove. 2005.

[38] Thorn CE, Bergesch C, Joyce A, Sambrano G, McDonnell K, Brennan F, et al. A robust, cost-effective method for DNA, RNA and protein co-extraction from soil, other complex microbiomes and pure cultures. Mol Ecol Resour. 2019;19:439–55. 30565880 10.1111/1755-0998.12979

[39] Joshi N, Fass J. Sickle: a sliding-window, adaptive, quality-based trimming tool for FastQ files (Version 1.33). Github;2011.

[40] Li D, Liu C-M, Luo R, Sadakane K, Lam T-W. MEGAHIT: an ultra-fast single-node solution for large and complex metagenomics assembly via succinct de Bruijn graph. Bioinformatics. 2015;31:1674–6. 25609793 10.1093/bioinformatics/btv033

[41] Uritskiy GV, DiRuggiero J, Taylor J. MetaWRAP—a flexible pipeline for genome-resolved metagenomic data analysis. Microbiome. 2018;6:1–13. 30219103 10.1186/s40168-018-0541-1 PMC6138922

[42] Parks DH, Imelfort M, Skennerton CT, Hugenholtz P, Tyson GW. CheckM: assessing the quality of microbial genomes recovered from isolates, single cells, and metagenomes. Genome Res. 2015;25:1043–55. 25977477 10.1101/gr.186072.114 PMC4484387

[43] Robbins SJ, Chan CX, Messer LF, Singleton CM, Geers AU, Ying H, et al. wwood/CoverM: read coverage calculator for metagenomics. Github;2017.

[44] Zhou Z, Tran PQ, Breister AM, Liu Y, Kieft K, Cowley ES, et al. METABOLIC: high-throughput profiling of microbial genomes for functional traits, metabolism, biogeochemistry, and community-scale functional networks. Microbiome. 2022;10:33. 35172890 10.1186/s40168-021-01213-8 PMC8851854

[45] Chaumeil P-A, Mussig AJ, Hugenholtz P, Parks DH. GTDB-Tk: a toolkit to classify genomes with the genome taxonomy database. Bioinformatics. 2019;36(6):1925–7. 31730192 10.1093/bioinformatics/btz848 PMC7703759

[46] Kanehisa M, Goto S. KEGG: Kyoto encyclopedia of genes and genomes. Nucleic Acids Res. 2000;28:27–30. 10592173 10.1093/nar/28.1.27 PMC102409

[47] Selengut JD, Haft DH, Davidsen T, Ganapathy A, Gwinn-Giglio M, Nelson WC, et al. TIGRFAMs and genome properties: tools for the assignment of molecular function and biological process in prokaryotic genomes. Nucleic Acids Res. 2007;35:D260–4. 17151080 10.1093/nar/gkl1043 PMC1781115

[48] Finn RD, Bateman A, Clements J, Coggill P, Eberhardt RY, Eddy SR, et al. Pfam: the protein families database. Nucleic Acids Res. 2014;42:D222–30. 24288371 10.1093/nar/gkt1223 PMC3965110

[49] Anantharaman K, Brown CT, Hug LA, Sharon I, Castelle CJ, Probst AJ, et al. Thousands of microbial genomes shed light on interconnected biogeochemical processes in an aquifer system. Nat Commun. 2016;7:13219. 27774985 10.1038/ncomms13219 PMC5079060

[50] Zhang H, Yohe T, Huang L, Entwistle S, Wu P, Yang Z, et al. dbCAN2: a meta server for automated carbohydrate-active enzyme annotation. Nucleic Acids Res. 2018;46:W95-101. 29771380 10.1093/nar/gky418 PMC6031026

[51] Rawlings ND, Barrett AJ, Finn R. Twenty years of the MEROPS database of proteolytic enzymes, their substrates and inhibitors. Nucleic Acids Res. 2016;44:D343–50. 26527717 10.1093/nar/gkv1118 PMC4702814

[52] Lee MD. GToTree: a user-friendly workflow for phylogenomics. Bioinformatics. 2019;35:4162–4. 30865266 10.1093/bioinformatics/btz188 PMC6792077

[53] Hug LA, Baker BJ, Anantharaman K, Brown CT, Probst AJ, Castelle CJ, et al. A new view of the tree of life. Nat Microbiol. 2016;1:1–6. 10.1038/nmicrobiol.2016.48 27572647

[54] Ashraf RJ. Multi-objective optimisation of anaerobic digestion systems PhD Thesis. Coventry University, UK. 2024.

[55] Katti S, Willems B, Meers E, Akyol C. Pilot-scale anaerobic digestion of on-farm agro-residues: boosting biogas production and digestate quality with thermophilic post-digestion. Waste Manag Bull. 2025;3(3):100201.

[56] Philippot L, Griffiths BS, Langenheder S. Microbial community resilience across ecosystems and multiple disturbances. Microbiol Mol Biol Rev. 2021;85(2):e00026-e120. 33789927 10.1128/MMBR.00026-20 PMC8139522

[57] Osburn ED, Yang G, Rillig MC. Evaluating the role of bacterial diversity in supporting soil ecosystem functions under anthropogenic stress. ISME Commun. 2023;3:66. 37400524 10.1038/s43705-023-00273-1 PMC10318037

[58] Qian JJ, Akçay E. The balance of interaction types determines the assembly and stability of ecological communities. Nat Ecol Evol. 2020;4:356–65. 32094535 10.1038/s41559-020-1121-x

[59] Muller EEL. Determining microbial niche breadth in the environment for better ecosystem fate predictions. mSystems. 2019. 10.1128/msystems.00080-19. 31186307 10.1128/mSystems.00080-19 PMC6584867

[60] De Vrieze J, Hennebel T, Boon N, Verstraete W. *Methanosarcina*: the rediscovered methanogen for heavy duty biomethanation. Bioresour Technol. 2012;112:1–9. 22418081 10.1016/j.biortech.2012.02.079

[61] Gunnigle E, Nielsen JL, Fuszard M, Botting CH, Sheahan J, O’Flaherty V, et al. Functional responses and adaptation of mesophilic microbial communities to psychrophilic anaerobic digestion. FEMS Microbiol Ecol. 2015;91(12):fiv132. 26507125 10.1093/femsec/fiv132

[62] Hardegen J, Latorre-Pérez A, Vilanova C, Günther T, Porcar M, Luschnig O, et al. Methanogenic community shifts during the transition from sewage mono-digestion to co-digestion of grass biomass. Bioresour Technol. 2018;265:275–81. 29906716 10.1016/j.biortech.2018.06.005

[63] Lü F, Hao L, Guan D, Qi Y, Shao L, He P. Synergetic stress of acids and ammonium on the shift in the methanogenic pathways during thermophilic anaerobic digestion of organics. Water Res. 2013;47:2297–306. 23434042 10.1016/j.watres.2013.01.049

[64] Yan M, Treu L, Zhu X, Tian H, Basile A, Fotidis IA, et al. Insights into ammonia adaptation and methanogenic precursor oxidation by genome-centric analysis. Environ Sci Technol. 2020;54:12568–82. 32852203 10.1021/acs.est.0c01945 PMC8154354

[65] Li C, Hao L, Lü F, Duan H, Zhang H, He P. Syntrophic acetate-oxidizing microbial consortia enriched from full-scale mesophilic food waste anaerobic digesters showing high biodiversity and functional redundancy. mSystems. 2022;7:e00339-22. 36073802 10.1128/msystems.00339-22 PMC9600251

