# Supplementary figures and images for: Using feeding regime as a microbial selective pressure to optimise biogas production and digestate sanitisation from slurry-based anaerobic digestion
Source: Environ Microbiome. 2026 May 22;21:92. doi: 10.1186/s40793-026-00902-x (PMC13404572; doi:10.1186/s40793-026-00902-x)

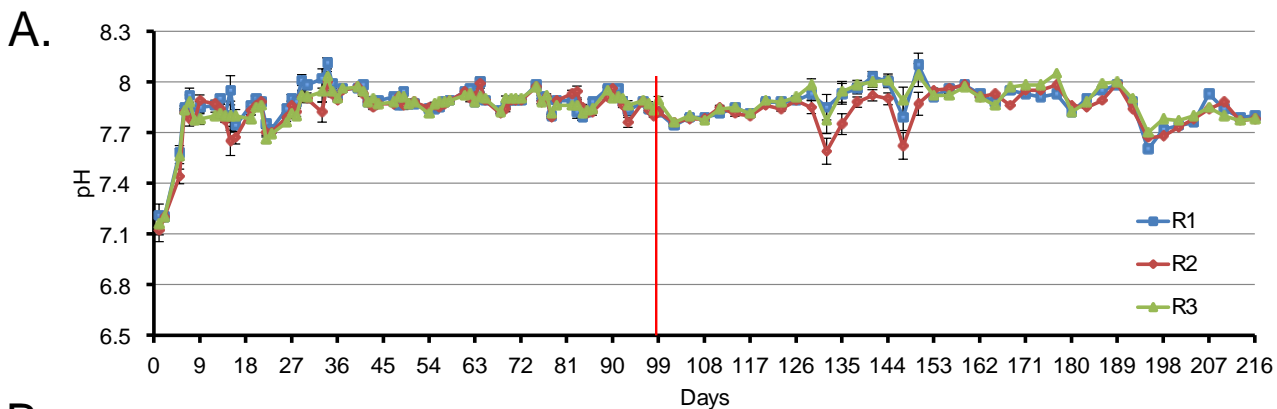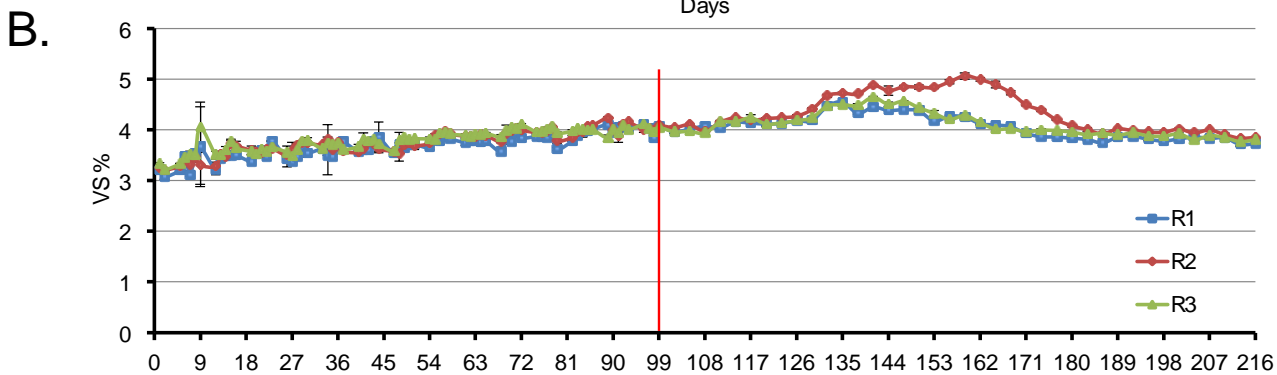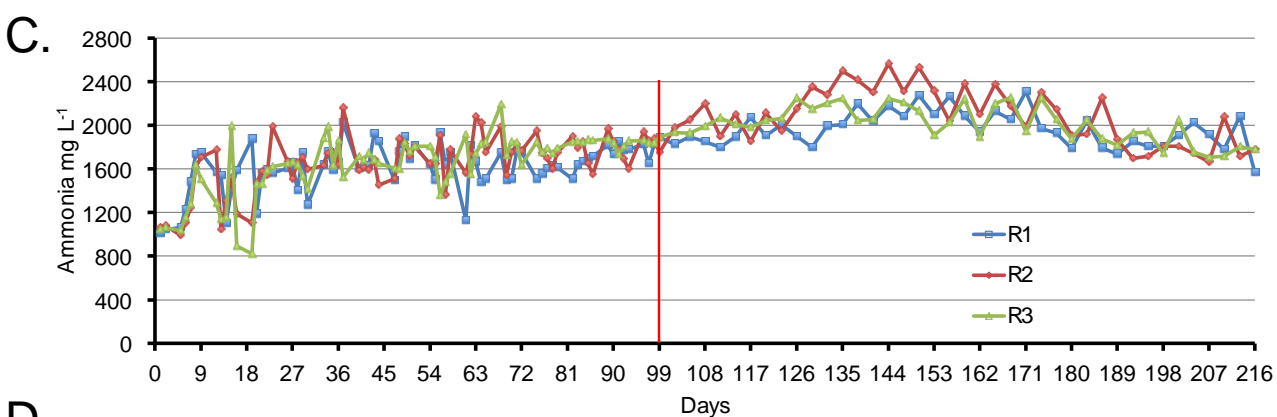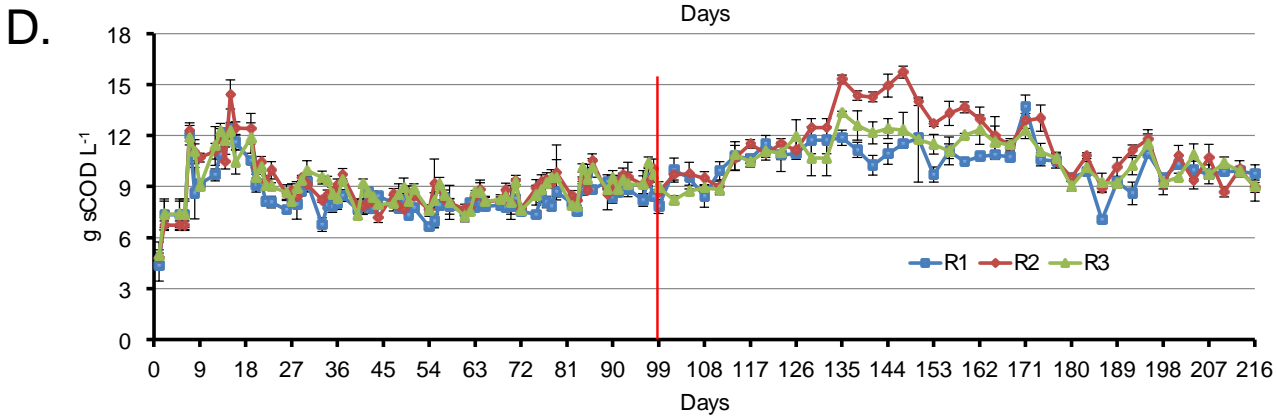

Supplement: Supplementary file 1 — Additional file 1: Physicochemical analysis of samples from triplicate CSTRs processing DCS and FOG for 216 days initially fed daily (first 99 days) and then fed every three days, including pH (A), volatile solids concentration (%; B), NH3 (C) and sCOD (D), with error bars indicating standard error of technical replicates (n = 3). A vertical red line indicates the change of feeding regime on Day 99. [file 40793_2026_902_MOESM1_ESM.pdf]

A.

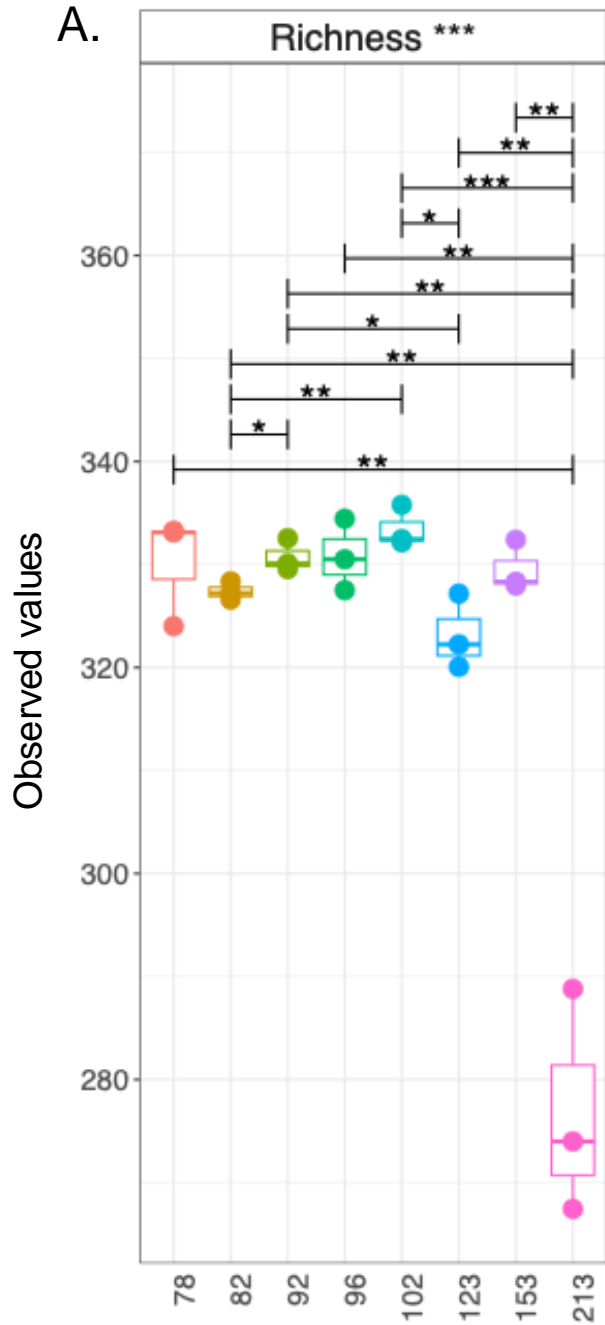

B.

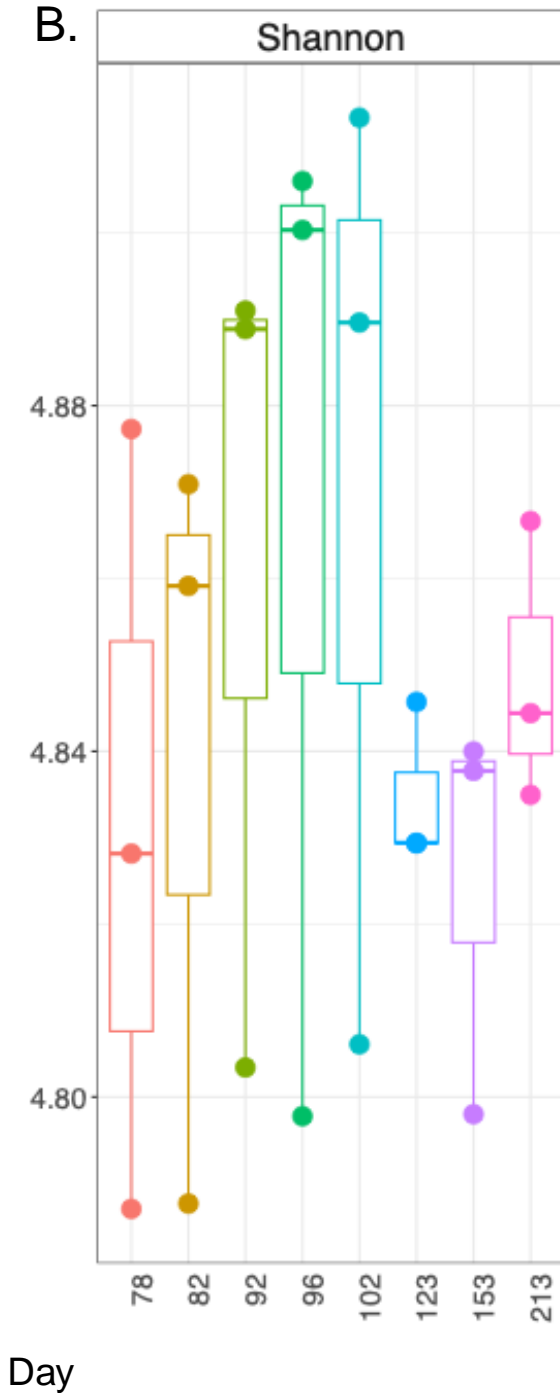

Supplement: Supplementary file 6 — Additional file 6: Novel bins. Phylogenetic gain (PG) was calculated using GTDBTK toolkit with higher values representing novelty of a particular genome within the context of the phylogenetic tree; the 10 most novel bins are shown. [file 40793_2026_902_MOESM6_ESM.pdf]

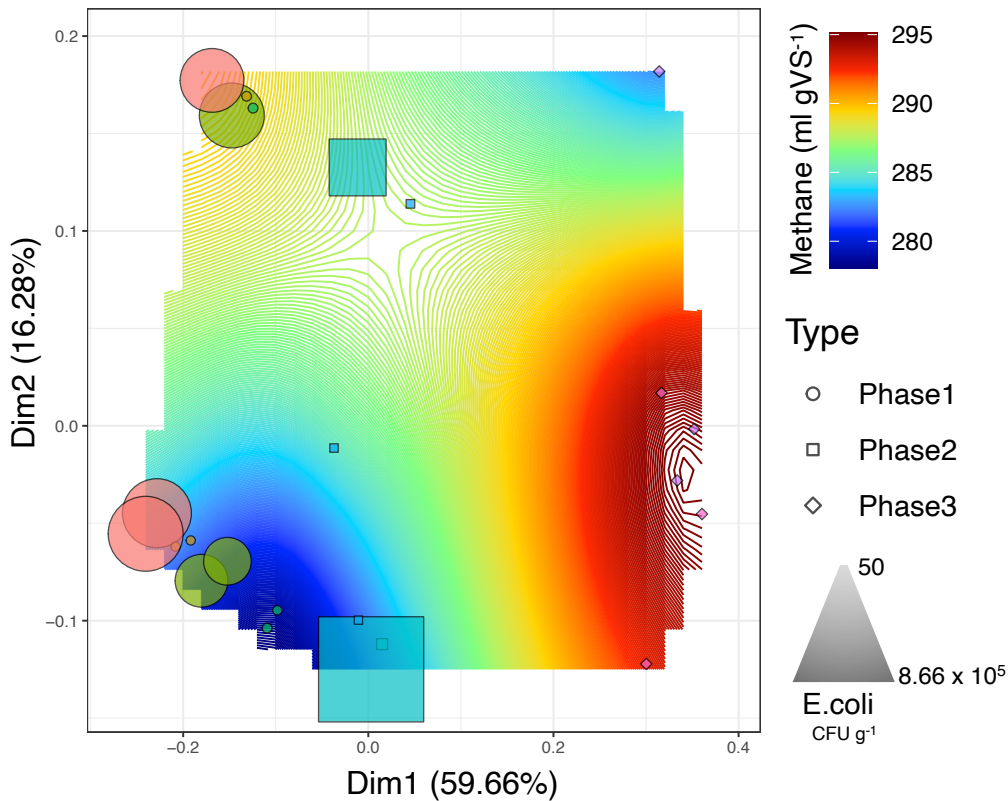

### Groups:

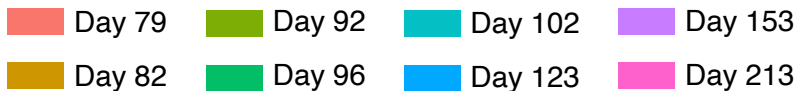

Supplement: Supplementary file 8 — Additional file 8: Microbial community structure shown as the relative abundances (proportions) of the Top-25 most abundant MAGS. [file 40793_2026_902_MOESM8_ESM.pdf]
