# Supplementary material for: Using feeding regime as a microbial selective pressure to optimise biogas production and digestate sanitisation from slurry-based anaerobic digestion
Source: Environ Microbiome. 2026 May 22;21:92. doi: 10.1186/s40793-026-00902-x (PMC13404572; doi:10.1186/s40793-026-00902-x)
Supplement: Supplementary file 2 — Additional file 2: Average physicochemical data of mixed feedstock throughout trial. [file 40793_2026_902_MOESM2_ESM.pdf]

Additional file 2: Average physicochemical data of mixed feedstock throughout trial

TS %	VS %	tCOD $g.L^{-1}$	sCOD $g.L^{-1}$	NH_3 $mg.L^{-1}$
10.02±0.06	7.78±0.04	165.78±8.38	20.40±0.56	1013.2±65.7
