# Supplementary material for: Using feeding regime as a microbial selective pressure to optimise biogas production and digestate sanitisation from slurry-based anaerobic digestion
Source: Environ Microbiome. 2026 May 22;21:92. doi: 10.1186/s40793-026-00902-x (PMC13404572; doi:10.1186/s40793-026-00902-x)
Supplement: Supplementary file 4 — Additional file 4: Microbial community structure shown as the relative abundance (proportions) of the archaeal fraction of the community. [file 40793_2026_902_MOESM4_ESM.pdf]

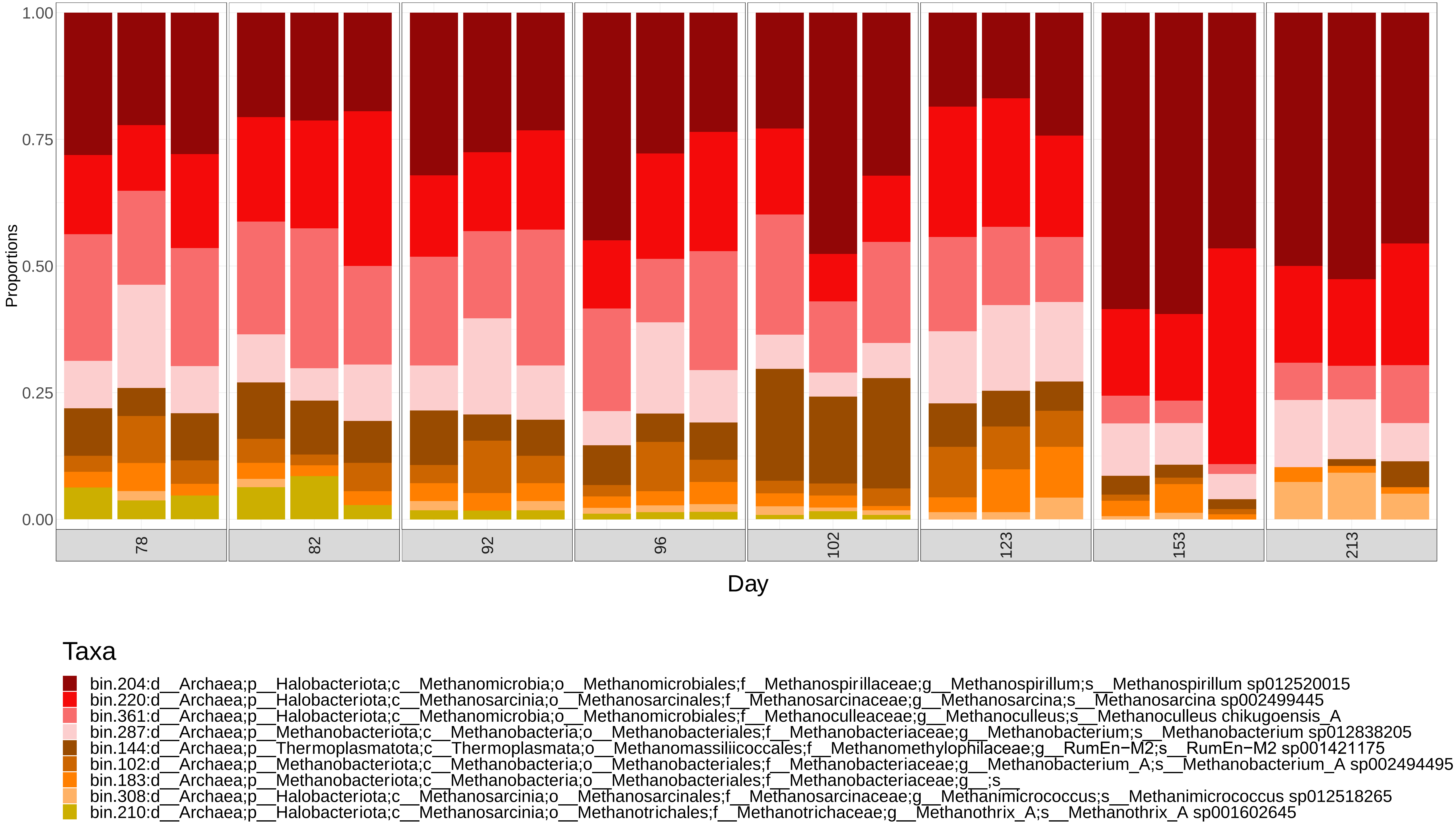
1.00
0.75
0.50
0.25
0.00
Proportions
78
82
92
96
102
123
153
213
Day
Taxa
bin.204:d__Archaea;p__Halobacteriota;c__Methanomicrobia;o__Methanomicrobiales;f__Methanospirillaceae;g__Methanospirillum;s__Methanospirillum sp012520015
bin.220:d__Archaea;p__Halobacteriota;c__Methanosarcinia;o__Methanosarcinales;f__Methanosarcinaceae;g__Methanosarcina;s__Methanosarcina sp002499445
bin.361:d__Archaea;p__Halobacteriota;c__Methanomicrobia;o__Methanomicrobiales;f__Methanoculleaceae;g__Methanoculleus;s__Methanoculleus chikugoensis_A
bin.287:d__Archaea;p__Methanobacteriota;c__Methanobacteria;o__Methanobacteriales;f__Methanobacteriaceae;g__Methanobacterium;s__Methanobacterium sp012838205
bin.144:d__Archaea;p__Thermoplasmatota;c__Thermoplasmata;o__Methanomassiliicoccales;f__Methanomethylophilaceae;g__RumEn−M2;s__RumEn−M2 sp001421175
bin.102:d__Archaea;p__Methanobacteriota;c__Methanobacteria;o__Methanobacteriales;f__Methanobacteriaceae;g__Methanobacterium_A;s__Methanobacterium_A sp002494495
bin.183:d__Archaea;p__Methanobacteriota;c__Methanobacteria;o__Methanobacteriales;f__Methanobacteriaceae;g__;s__
bin.308:d__Archaea;p__Halobacteriota;c__Methanosarcinia;o__Methanosarcinales;f__Methanosarcinaceae;g__Methanimicrococcus;s__Methanimicrococcus sp012518265
bin.210:d__Archaea;p__Halobacteriota;c__Methanosarcinia;o__Methanotrichales;f__Methanotrichaceae;g__Methanothrix_A;s__Methanothrix_A sp001602645
