# Supplementary material for: Using feeding regime as a microbial selective pressure to optimise biogas production and digestate sanitisation from slurry-based anaerobic digestion
Source: Environ Microbiome. 2026 May 22;21:92. doi: 10.1186/s40793-026-00902-x (PMC13404572; doi:10.1186/s40793-026-00902-x)
Supplement: Supplementary file 5 — Additional file 5: E. coli removal and methane production. E. coli values were fit on the first two dimensions of the ordination plot using smooth cubic splines (CS) interpolation after fitting generalised additive model of the form E. coli ~ CS (Dim1, Dim2), which returned as significant with p<0.05; phases are represented by shape, size of the shape represents the quantity of E. coli and the heatmap shows the quantity of methane generated at those timepoints. [file 40793_2026_902_MOESM5_ESM.pdf]

Taxon	PG	Phylum	Last defined rank
bin.96	1.082	*Patescibacteria*	c__*Dojkabacteria*
bin.366	0.908	*Campylobacterota*	f__*Arcobacteraceae*
bin.216	0.741	*Fibrobacterota*	f__*Fibrobacteraceae*
bin.265	0.720	*Patescibacteria*	p__*Patescibacteria*
bin.239	0.708	*Planctomycetota*	f__*Pirellulaceae*
bin.233	0.697	*Patescibacteria*	o__*Paceibacterales*
bin.351	0.657	*Caldatribacteriota*	o__*Caldatribacteriales*
bin.151	0.615	*Myxococcota*	p__*Myxococcota*
bin.243	0.580	*Acidobacteriota*	p__*Acidobacteriota*
bin.193	0.549	*Actinobacteriota*	c__*Thermoleophilia*
