# Supplementary material for: Using feeding regime as a microbial selective pressure to optimise biogas production and digestate sanitisation from slurry-based anaerobic digestion
Source: Environ Microbiome. 2026 May 22;21:92. doi: 10.1186/s40793-026-00902-x (PMC13404572; doi:10.1186/s40793-026-00902-x)
Supplement: Supplementary file 7 — Additional file 7: Functional microbial community diversity measured as (A) richness and (B) Shannon entropy of the recovered KEGG Modules (KEGG Module Hit), coloured according to day of sample collection. [file 40793_2026_902_MOESM7_ESM.pdf]

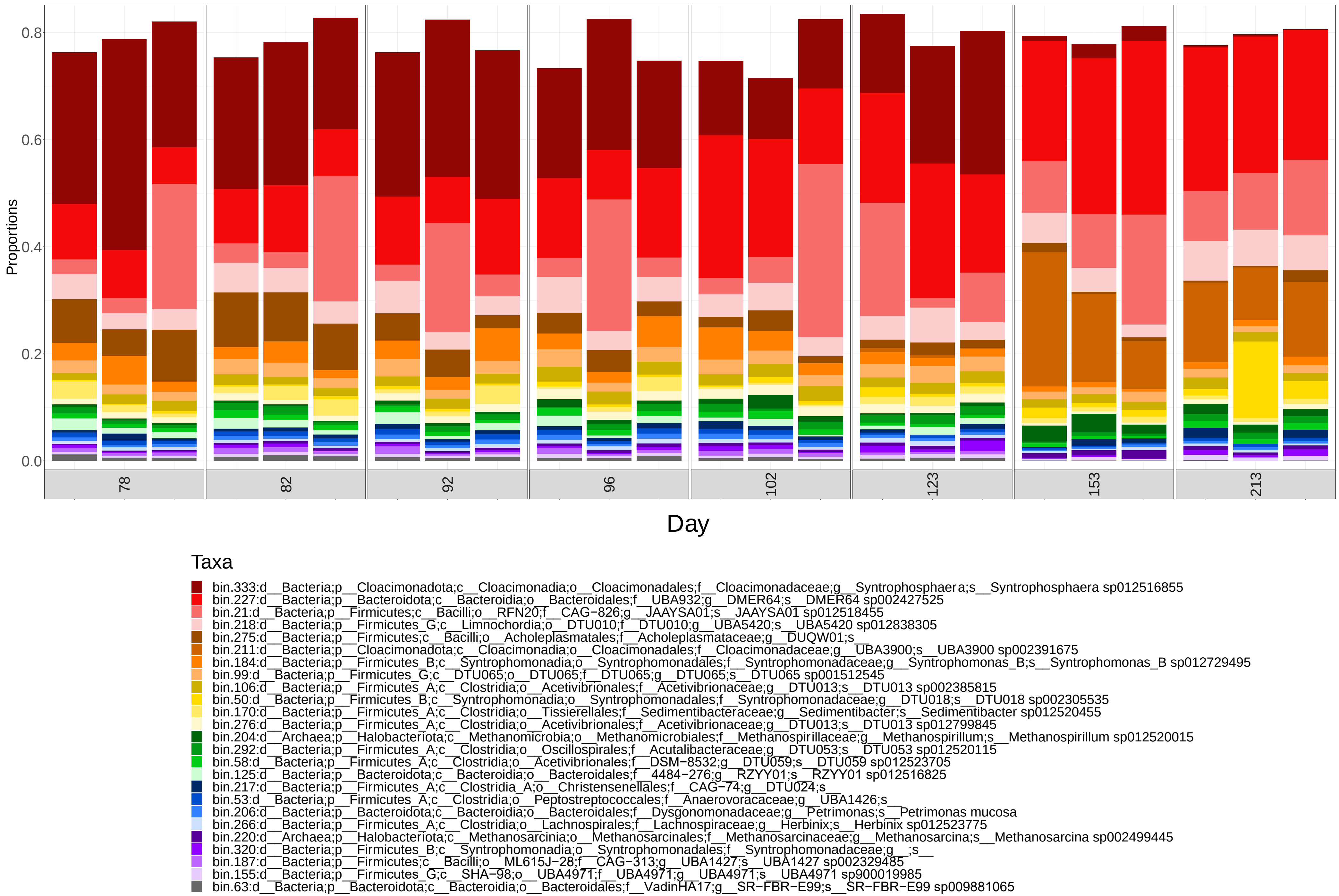
0.8
0.6
0.4
0.2
0.0
Proportions
78
82
92
96
102
123
153
213
Day
Taxa
bin.333:d__Bacteria;p__Cloacimonadota;c__Cloacimonadia;o__Cloacimonadales;f__Cloacimonadaceae;g__Syntrophosphaera;s__Syntrophosphaera sp012516855
bin.227:d__Bacteria;p__Bacteroidota;c__Bacteroidia;o__Bacteroidales;f__UBA932;g__DMER64;s__DMER64 sp002427525
bin.21:d__Bacteria;p__Firmicutes;c__Bacilli;o__RFN20;f__CAG−826;g__JAAYSA01;s__JAAYSA01 sp012518455
bin.218:d__Bacteria;p__Firmicutes_G;c__Limnochordia;o__DTU010;f__DTU010;g__UBA5420;s__UBA5420 sp012838305
bin.275:d__Bacteria;p__Firmicutes;c__Bacilli;o__Acholeplasmatales;f__Acholeplasmataceae;g__DUQW01;s__
bin.211:d__Bacteria;p__Cloacimonadota;c__Cloacimonadia;o__Cloacimonadales;f__Cloacimonadaceae;g__UBA3900;s__UBA3900 sp002391675
bin.184:d__Bacteria;p__Firmicutes_B;c__Syntrophomonadia;o__Syntrophomonadales;f__Syntrophomonadaceae;g__Syntrophomonas_B;s__Syntrophomonas_B sp012729495
bin.99:d__Bacteria;p__Firmicutes_G;c__DTU065;o__DTU065;f__DTU065;g__DTU065;s__DTU065 sp001512545
bin.106:d__Bacteria;p__Firmicutes_A;c__Clostridia;o__Acetivibrionales;f__Acetivibrionaceae;g__DTU013;s__DTU013 sp002385815
bin.50:d__Bacteria;p__Firmicutes_B;c__Syntrophomonadia;o__Syntrophomonadales;f__Syntrophomonadaceae;g__DTU018;s__DTU018 sp002305535
bin.170:d__Bacteria;p__Firmicutes_A;c__Clostridia;o__Tissierellales;f__Sedimentibacteraceae;g__Sedimentibacter;s__Sedimentibacter sp012520455
bin.276:d__Bacteria;p__Firmicutes_A;c__Clostridia;o__Acetivibrionales;f__Acetivibrionaceae;g__DTU013;s__DTU013 sp012799845
bin.204:d__Archaea;p__Halobacteriota;c__Methanomicrobia;o__Methanomicrobiales;f__Methanospirillaceae;g__Methanospirillum;s__Methanospirillum sp012520015
bin.292:d__Bacteria;p__Firmicutes_A;c__Clostridia;o__Oscillospirales;f__Acutalibacteraceae;g__DTU053;s__DTU053 sp012520115
bin.58:d__Bacteria;p__Firmicutes_A;c__Clostridia;o__Acetivibrionales;f__DSM−8532;g__DTU059;s__DTU059 sp012523705
bin.125:d__Bacteria;p__Bacteroidota;c__Bacteroidia;o__Bacteroidales;f__4484−276;g__RZYY01;s__RZYY01 sp012516825
bin.217:d__Bacteria;p__Firmicutes_A;c__Clostridia_A;o__Christensenellales;f__CAG−74;g__DTU024;s__
bin.53:d__Bacteria;p__Firmicutes_A;c__Clostridia;o__Peptostreptococcales;f__Anaerovoracaceae;g__UBA1426;s__
bin.206:d__Bacteria;p__Bacteroidota;c__Bacteroidia;o__Bacteroidales;f__Dysgonomonadaceae;g__Petrimonas;s__Petrimonas mucosa
bin.266:d__Bacteria;p__Firmicutes_A;c__Clostridia;o__Lachnospirales;f__Lachnospiraceae;g__Herbinix;s__Herbinix sp012523775
bin.220:d__Archaea;p__Halobacteriota;c__Methanosarcinia;o__Methanosarcinales;f__Methanosarcinaceae;g__Methanosarcina;s__Methanosarcina sp002499445
bin.320:d__Bacteria;p__Firmicutes_B;c__Syntrophomonadia;o__Syntrophomonadales;f__Syntrophomonadaceae;g__;s__
bin.187:d__Bacteria;p__Firmicutes;c__Bacilli;o__ML615J−28;f__CAG−313;g__UBA1427;s__UBA1427 sp002329485
bin.155:d__Bacteria;p__Firmicutes_G;c__SHA−98;o__UBA4971;f__UBA4971;g__UBA4971;s__UBA4971 sp900019985
bin.63:d__Bacteria;p__Bacteroidota;c__Bacteroidia;o__Bacteroidales;f__VadinHA17;g__SR−FBR−E99;s__SR−FBR−E99 sp009881065
