# Supplementary material for: Using feeding regime as a microbial selective pressure to optimise biogas production and digestate sanitisation from slurry-based anaerobic digestion
Source: Environ Microbiome. 2026 May 22;21:92. doi: 10.1186/s40793-026-00902-x (PMC13404572; doi:10.1186/s40793-026-00902-x)
Supplement: Supplementary file 9 — Additional file 9: PERMANOVA results for key environmental covariates where R2 explains percentage variability (scaled to 1) when significant (p ≤ 0.05). Each abundance table is an N samples x P features table mainly from METABOLIC software with the Bray-Curtis distance between samples supplied to the PERMANOVA procedure along with the covariate’s data. Here N.S. represents non-significant results. [file 40793_2026_902_MOESM9_ESM.pdf]

Additional file 7: PERMANOVA results for key environmental covariates where R^2 explains percentage variability (scaled to 1) when significant ($p \leq 0.05$). Each abundance table is an N samples x P features table mainly from METABOLIC software with the Bray-Curtis distance between samples supplied to the PERMANOVA procedure along with the covariate's data. Here N.S. represents non-significant results.

**Covariate**	**Microbial community composition**	**METABOLIC Recovered Functions**					
		**dbCAN2 (CAZy)**	**HMM**	**Function**	**KEGG Module**	**KEGG Module Steps**	**MEROPS (Peptidase)**
Day	R^2 = 0.50762 (*p* = 0.0001, ***)	R^2 = 0.1713 (*p* = 0.0035, **)	R^2 = 0.2195 (*p* = 0.0005, ***)	R^2 = 0.19109 (*p* = 0.0026, **)	R^2 = 0.20115 (*p* = 0.0036, **)	R^2 = 0.1327 (*p* = 0.0334, *)	R^2 = 0.28641 (*p* = 0.0001, ***)
Phase	R^2 = 0.62278 (*p* = 0.0001, ***)	R^2 = 0.27796 (*p* = 0.0002, ***)	R^2 = 0.28163 (*p* = 0.0001, ***)	R^2 = 0.25553 (*p* = 0.0013, **)	R^2 = 0.24435 (*p* = 0.0041, **)	N.S.	R^2 = 0.39873 (*p* = 0.0001, ***)
Reactor	N.S.	R^2 = 0.27163 (*p* = 0.0004, ***)	R^2 = 0.21697 (*p* = 0.003, **)	R^2 = 0.23948 (*p* = 0.0013, **)	R^2 = 0.24612 (*p* = 0.0039, **)	R^2 = 0.26182 (*p* = 0.0036, **)	R^2 = 0.17985 (*p* = 0.0359, *)
Methane	N.S.	N.S.	N.S.	N.S.	N.S.	N.S.	N.S.
Ammonia	R^2 = 0.19723 (*p* = 0.0042, **)	R^2 = 0.13311 (*p* = 0.012, *)	R^2 = 0.12735 (*p* = 0.0141, *)	R^2 = 0.11586 (*p* = 0.0261, *)	N.S.	N.S.	R^2 = 0.17514 (*p* = 0.0037, **)
E. coli	R^2 = 0.33528 (*p* = 0.0001, ***)	R^2 = 0.11363 (*p* = 0.0273, *)	R^2 = 0.13434 (*p* = 0.0156, *)	R^2 = 0.11291 (*p* = 0.0304, *)	N.S.	N.S.	R^2 = 0.1995 (*p* = 0.0013, **)
